# Global end-diastolic volume increases to maintain fluid responsiveness in sepsis-induced systolic dysfunction

**DOI:** 10.1186/1471-2253-13-12

**Published:** 2013-06-22

**Authors:** Ronald J Trof, Ibrahim Danad, AB Johan Groeneveld

**Affiliations:** 1 Department of Intensive Care, Vrije Universiteit Medical Centre, Amsterdam, The Netherlands; 2 Department of Intensive Care, Medisch Spectrum Twente, Enschede, The Netherlands; 3 Department of Intensive Care, Erasmus Medical Centre, Rotterdam, The Netherlands

**Keywords:** Septic Shock, Fluid Loading, Myocardial Depression, Right Ventricular Afterload, Diastolic Compliance, Filling Volumes, Ejection Fraction

## Abstract

**Background:**

Sepsis-induced cardiac dysfunction may limit fluid responsiveness and the mechanism thereof remains unclear. Since cardiac function may affect the relative value of cardiac filling pressures, such as the recommended central venous pressure (CVP), versus filling volumes in guiding fluid loading, we studied these parameters as determinants of fluid responsiveness, according to cardiac function.

**Methods:**

A delta CVP-guided, 90 min colloid fluid loading protocol was performed in 16 mechanically ventilated patients with sepsis-induced hypotension and three 30 min consecutive fluid loading steps of about 450 mL per patient were evaluated. Global end-diastolic volume index (GEDVI), cardiac index (CI) and global ejection fraction (GEF) were assessed from transpulmonary dilution. Baseline and changes in CVP and GEDVI were compared among responding (CI increase ≥10% and ≥15%) and non-responding fluid loading steps, in patient with low (<20%, n = 9) and near-normal (≥20%) GEF (n = 7) at baseline.

**Results:**

A low GEF was in line with other indices of impaired cardiac (left ventricular) function, prior to and after fluid loading. Of 48 fluid loading steps, 9 (of 27) were responding when GEF <20% and 6 (of 21) when GEF ≥20. Prior to fluid loading, CVP did not differ between responding and non-responding steps and levels attained were 23 higher in the latter, regardless of GEF (P = 0.004). Prior to fluid loading, GEDVI (and CI) was higher in responding (1007 ± 306 mL/m^2^) than non-responding steps (870 ± 236 mL/m^2^) when GEF was low (P = 0.002), but did not differ when GEF was near-normal. Increases in GEDVI were associated with increases in CI and fluid responsiveness, regardless of GEF (P < 0.001).

**Conclusions:**

As estimated from transpulmonary dilution, about half of patients with sepsis-induced hypotension have systolic cardiac dysfunction. During dysfunction, cardiac dilation with a relatively high baseline GEDVI maintains fluid responsiveness by further dilatation (increase in GEDVI rather than of CVP) as in patients without dysfunction. Absence of fluid responsiveness during systolic cardiac dysfunction may be caused by diastolic dysfunction and/or right ventricular dysfunction.

## Background

Patients with severe sepsis or septic shock commonly develop cardiac dysfunction, even in the absence of cardiac ischemia [1-3]. These abnormalities may include depression of left and/or right ventricular systolic function and/or diastolic dysfunction and may be accompanied by ventricular dilatation, as estimated from echocardiography or radionuclide cineangiography [4,5]. This cardiac dysfunction is usually reversible and returns to normal in 7 to 10 days in survivors [6-8]. Systolic dysfunction-induced ventricular dilatation is suggested to be an adaptive mechanism to maintain a high cardiac output which is associated with survival [4,9], while other investigators denied such a dilatory response arguing in favor of impaired relaxation and diastolic (often upon systolic) dysfunction contributing to non-survival [8,10-16].

Fluid loading is often the initial treatment of sepsis-induced hypotension and the response may be diminished in sepsis-induced cardiac depression associated with severe disease and non-survival (5,13,14). On the other hand, fluid overloading when the heart is non-responsive and the central venous pressure (CVP) is inadvertently elevated is potentially harmful and also associated with mortality, emphasizing the value of appropriate haemodynamic monitoring [17]. By optimizing preload and assessing fluid responsiveness, deleterious hypoperfusion and fluid overloading may be prevented. Traditionally, filling pressures, like CVP, have been used to guide fluid loading in sepsis-induced hypotension [17-20], even though its predictive value for fluid responsiveness during mechanical ventilation and altered cardiac function is doubtful [21-23]. Alternatively, the transpulmonary dilution technique estimates the global end-diastolic volume index (GEDV), and pulmonary blood volume index (PBVI) as a superior and global measures of cardiac preload [11,23,24]. The GEDVI represents the volumes of the right and left heart at the end of diastole and often reflects left ventricular end-diastolic volume estimated by echocardiography provided that right ventricular dilatation is absent [25].

A relatively low GEDVI may predict fluid responsiveness (and a relatively high GEDVI absence thereof), but the role of systolic and/or diastolic dysfunction with respect to interpretation of absolute values remains unclear, even though changes in stroke volume or cardiac output correlate to changes in GEDVI [21,23,26]. Indeed, the relative value of GEDVI and filling pressures in determining fluid responsiveness depends on systolic cardiac function, at least in non-septic patients [27]. Conversely, echocardiographic end-diastolic left ventricular dimensions poorly predicted fluid responsiveness but changes were superior to filling pressures in monitoring changes in cardiac output upon fluid loading in some studies on sepsis [9,23]. In contrast, fluid responsiveness was found to be associated with biventricular dilatation by nuclear angiography and non-responsiveness appeared attributable to right ventricular systolic dysfunction following mild pulmonary hypertension in other studies on sepsis [6,9].

In view of the above controversies on mechanisms and predictive values, we evaluated and compared filling volumes to pressures in determining the cardiac response to fluid loading according to systolic cardiac function in sepsis-induced hypotension, in the hypothesis that, even in dysfunctioning hearts, cardiac dilatation is required to increase cardiac output upon fluid loading.

### Methods

This was a sub-study of a prospective, non-randomized, single-center clinical trial, investigating the cardiorespiratory effects of various resuscitation fluids in presumed hypovolemia during sepsis and non-sepsis, in mechanically ventilated patients in the intensive care unit (ICU) [24,28]. We analyzed, retrospectively, 16 patients with sepsis monitored by both CVP and the transpulmonary dilution technique. These patients were divided in two groups according to a low GEF (<20%) and near-normal GEF (≥20%). The cutoff of 20% approximately reflects a cutoff of 40% ejection fraction of the left ventricle as measured by echocardiography, provided that there is no right ventricular dysfunction [29-31]. The original study was approved by the Ethics Committee of the Vrije Universiteit Medical Center and written informed consent was obtained. We analyzed the effect of colloid fluid loading in patients with sepsis-induced hypotension. Colloid fluid loading was given with modified fluid gelatin 4%, hydroxyethyl starch (HES) 6% or albumin 5%, all of which have similar oncotic properties and haemodynamic responses [24,28,32]. We only analyzed patients who completed fluid loading and measurements up to t = 90 min. Inclusion criteria, at enrollment and start of the protocol, were clinical criteria for presumed hypovolemia commonly triggering fluid infusion, such as a relatively arbitrarily chosen systolic blood pressure <110 mmHg and a low CVP, roughly taking transmission of positive end-expiratory pressure (PEEP) into account (Table 1). Exclusion criteria were age >75 year, preterminal illness with a life expectancy of less than 24 hours, or known anaphylactic reactions to colloids. Sepsis was defined according to international guidelines [33]. The origin of sepsis was defined by clinical signs and symptoms, imaging techniques and positive local and/or blood cultures [33]. All patients were on controlled mechanical ventilation and positive end-expiratory pressure (PEEP).

**Table 1:** Fluid challenge protocol

CVP at start:	≤ 8 if PEEP ≤15	200 ml/10 min
	≤ 12 if PEEP >15	200 ml/10 min
	≤ 10 if PEEP ≤15	100 ml/10 min
	≤ 14 if PEEP >15	100 ml/10 min
	≤ 12 if PEEP ≤15	50 ml/10 min
	≤ 16 if PEEP >15	50 ml/10 min
CVP during infusion:	increase >5	stop
CVP after 10 min:	increase ≤2	continue
	2< increase ≤5	wait 10 min
	increase >5	stop
CVP after 10 min waiting:	increase >2	stop
	increase ≤2	repeat

*CVP* Central venous pressure (mmHg), *PEEP* Positive end-expiratory pressure (cm H_2_O).

### Study protocol

The protocol was started in the ICU when patients met the inclusion criteria. Demographic characteristics were recorded, including the acute physiology and chronic health evaluation (APACHE-II). After baseline measurements were taken, fluids were given over 90 min on the basis of the response within predefined limits of increases in CVP, according to a previously described protocol (Table 1)[24,28,32]. Up to 200 mL of fluid were given every 10 min, provided that the increase in CVP upon fluid loading did not exceed critical values, and this policy has been proven safe in previous studies (i.e. not evoking pulmonary edema)[24,28]. The maximum amount of fluid infused was 1800 mL. Fluid responsiveness was defined as an increase of CI ≥10 and 15%, in accordance with the literature [22,33], between t = 0-30, t = 30-60 and t = 60-90 min upon fluid loading. Concomitant vasoactive and sedative drug treatment and ventilatory settings remained unchanged during fluid loading.

### Measurements

Heart rate (HR) and mean arterial pressure (MAP) were recorded at t = 0 and 90 min. MAP and CVP were measured in the supine position after calibration, zeroing to atmospheric pressure at the midchest level at end-expiration (Tramscope^R^, Marquette GE, Milwaukee, Wisconsin). Cardiac output, GEDVI, PBVI and CVP were measured every 30 min, from t = 0 to 90 min. Relevant measurements were indexed to body surface area (BSA), giving stroke volume index (SVI, mL/m^2^), cardiac index (CI, L/min/m^2^), GEDVI (n 680–800 mL/m^2^) and PBVI (n 150–250 mL/m^2^), respectively. For these measurements, the transpulmonary thermal-dye indicator dilution technique was used (11). These measurements involve averages obtained from 2–3 central venous injections of 15 mL of ice-cold indocyanine green in 5% glucose solution and concomitant registration of the dilution curves in the femoral artery, by a 3 F catheter equipped with a thermistor (PV 2024, Pulsion Medical Systems, Munich, Germany). This catheter was inserted via a 4 F introducing sheath (Arrow, Reading, USA) and connected to a bedside computer (COLD Z-021, Pulsion Medical Systems, Munich, Germany. The COLD Z-021 is the precursor to the current transpulmonary thermodilution pulse contour cardiac output (PiCCO™) technique and yields the same cardiac parameters. Reproducibility of measurements is typically within 10% (11). GEDVI represents the volumes of the right and left heart at end-diastole and reflects left ventricular dimensions obtained by echocardiography in the absence of overt right ventricular distention (25). The ratio between stroke volume index (cardiac index/HR) and GEDVI/4 is defined as the global ejection fraction (GEF, normal values 25-35%), and is an indicator of left ventricular systolic function, provided that there is no right ventricular dysfunction (29–31). Left ventricular stroke work index (LVSWI, gm/m^2^) was calculated from SVI x (MAP-CVP) x 0.0136 and cardiac function index (CFI, n 18.0-26.0 1/min) from CI/(GEDVI/4) (30,31). Preload-recruitable stroke work was defined by LVSWI/GEDVI (24). CFI, LVSWI and LVSWI/GEDVI were used to assess cardiac (e.g. left ventricular) systolic function. The lung injury score was calculated from radiographic densities, oxygenation ratio P_a_O_2_/F_I_O_2_, PEEP and dynamic compliance and ranges between 0–4. Mortality refers to death in the ICU.

### Statistical analysis

For categorical data, Fisher exact tests were used. Since continuous data were normally distributed (Kolmogorov-Smirnov test, *P* >0.05), they were summarized by mean ± standard deviation (SD) and parametric tests were done. Paired and unpaired t-tests were used to compare data in time and between GEF groups, respectively generalized estimating equations (GEE) were used to evaluate differences in baseline and changes in variables between summated responding and non-responding fluid loading steps in each GEF group, to evaluate their determining values, respectively, taking repeated measurements in the same patients and type and volume of fluid administered (as covariates) into account. Exact two-sided P values >0.001 are given and considered statistically significant when <0.05. All analyses were conducted using SPSS version 15.0 (SPSS Chicago, Ill, USA).

## Results

Table 2 summarizes the characteristics of patients. The haemodynamic variables differ according to GEF and changes upon fluid loading. There was no difference in the amount and type of fluids infused between the GEF groups. GEF did not change during fluid loading. In the low GEF group, other function indices also pointed to systolic cardiac dysfunction, prior to and after fluid loading, even though the CI attained with fluid loading did not differ among the groups. The number of fluid loading responses did not differ according to GEF, but the increase in CI decreased with increasing fluid loading steps only when GEF was low (P = 0.04). The increases with fluids in CVP, GEDVI, MAP, LVSWI and CI did not differ among GEF groups, even though SVI, PBVI and LVSWI/GEDVI increased in the low GEF group only.

**Table 2:** Patient characteristics

	**GEF <20%**	**GEF ≥20%**	
	(n = 9)	(n = 7)	*P* value
Age	62 ± 9	57 ± 9	0.32
Male/female	7/2	4/3	0.60
APACHE II	16 ± 4	12 ± 5	0.08
Cardiac premorbidity	4	1	0.31
Sepsis origin			0.38
pulmonary	4	3	
abdominal	2	0	
CNS	0	1	
urogenital	1	0	
unknown	2	3	
Bloodstream infection			0.41
Gram-	2		
Gram+	2	2	
Fungi		1	
PEEP, cm H_2_O	14 ± 6	12 ± 3	0.17
Tidal volume, mL/kg	8.0 ± 0.8	9.0 ± 1.6	0.08
P_a_O_2_/F_I_O_2_	209 ± 54	193 ± 62	0.60
Lung injury score	2.2 ± 0.8	2.5 ± 0.8	0.60
ICU mortality	4	2	0.37
**Haemodynamics**			
HR, /min			
t = 0	106 ± 18	90 ± 25	0.15
t = 90	103 ± 16	95 ± 22	0.42
MAP, mm Hg			
t = 0	73 ± 12	74 ± 9	0.84
t = 90	88 ± 19^1^	89 ± 13^1^	0.87
CVP, mm Hg			
t = 0	9 ± 5	8 ± 3	0.61
t = 90	12 ± 5^2^	12 ± 3^2^	0.83
CI, L/min			
t = 0	3.3 ± 0.6	4.3 ± 1.5	0.06
t = 90	3.9 ± 1.0^2^	5.0 ± 1.4^3^	0.09
SVI, mL/m^2^			
t = 0	31 ± 6	49 ± 12	0.002
t = 90	38 ± 9^1^	53 ± 11	0.01
LVSWI, gm/m^2^			
t = 0	27 ± 5	43 ± 8	<0.001
t = 90	39 ± 11^3^	55 ± 11^4^	0.01
GEDVI, mL/m^2^			
t = 0	891 ± 257	787 ± 140	0.35
t = 90	963 ± 273^1^	866 ± 170^1^	0.43
GEF,%			
t = 0	15 ± 2	25 ± 5	n.a.
t = 90	16 ± 4	25 ± 7	0.005
CFI, 1/min			
t = 0	15.2 ± 2.9	22.1 ± 6.2	0.01
t = 90	16.8 ± 4.0	23.4 ± 5.5	0.01
LVSWI/GEDVI, gm/mL			
t = 0	0.13 ± 0.04	0.22 ± 0.04	<0.001
t = 90	0.17 ± 0.07^4, 3^	0.26 ± 0.07	0.02
Norepinephrine and/or dopamine	8	6	0.70
Norepinephrine, μg/kg/min	0.09 ± 0.11	0.06 ± 0.12	0.25
Dopamine, μg/kg/min	5.6 ± 3.4	4.9 ± 4.3	0.92
Fluid, mL	1456 ± 296	1271 ± 269	0.22
Gelatin/HES/albumin	3/2/4	2/3/2	0.66

Mean ± SD or number of patients, where appropriate.

Abbreviations: *GEF* Global ejection fraction, *APACHE II* Acute physiology and chronic health evaluation, *CNS* Central nervous system, *PEEP* Positive end-expiratory pressure, *ICU* Intensive care unit, *P*_*a*_*O*_*2*_*/F*_*I*_*O*_*2*_ Arterial partial pressure of O_2_ over inspiratory O_2_ fraction, *HR* Heart rate, *MAP* Mean arterial pressure, *CVP* Central venous pressure, *CI* Cardiac index, *SVI* Stroke volume index, *LVSWI* Left ventricular stroke volume index, *GEDVI* Global end-diastolic volume, *CFI* Cardiac function index, *HES* Hydroxyethyl starch. ^1^*P* = 0.02 , ^2^*P* = 0.002, ^3^P = 0.009, ^3^*P* = 0.04 vs. t = 0, *n.a.* not applicable.

### Fluid loading steps in GEF groups

Responses were independent of the type of colloid fluid, regardless of GEF and cutoff for fluid responsiveness. The CI prior to each fluid loading step was higher in responding than non-responding steps in the low GEF group, but lower in the near-normal GEF group (Table 3). The CVP did not differ between responding and non-responding steps in both GEF groups but attained higher values after fluid loading in non-responding than in responding steps, regardless of GEF. When GEF was low, GEDVI was higher prior to responding than non-responding fluid loading steps, while GEDVI in the near-normal GEF group did not differ prior to fluid loading steps. The GEDVI and PBVI increased in responding fluid loading steps regardless of GEF. Hence, baseline CVP and GEDVI were poor predictors of fluid responsiveness in both GEF groups. When fluid responsiveness was defined as an increase in CI ≥15%, changes in CO were also directly associated with changes in GEDVI, but not in PBVI. Otherwise there were only 4 out of 9 responding steps remaining when defining fluid responsiveness by 15 vs. 10% CI increases, in patients with low GEF needing relatively large amounts of fluid. Baseline GEDVI was not lower in responders than non-responders.

**Table 3:** Summated fluid loading steps, with responsiveness defined as ≥10% and ≥15% increase in cardiac index (CI), when systolic cardiac function is reduced or near-normal at 20% cutoff of global ejection fraction (GEF)

	**GEF <20% (n = 9)**			**GEF ≥20% (n = 7)**		
**CI ≥10%**	**R**	**NR**	**P-value**	**R**	**NR**	**P-value**
	**(n = 9 steps in 6 patients)**	**(n = 18 steps in 9 patients)**		**(n = 6 stepsin 5 patients)**	**(n = 15 steps in 7 patients)**	
CI, L/min/m^2^						
baseline	3.7 ± 0.7	3.5 ± 0.7	0.04	3.6 ± 1.2	5.0 ± 1.5	0.008
after	4.4 ± 0.8	3.4 ± 0.6		4.3 ± 1.4	5.0 ± 1.5	
change	0.7 ± 0.3	0 ± 0.3	n.a.	0.7 ± 0.3	0 ± 0.2	n.a.
CVP, mm Hg						
baseline	9 ± 6	11 ± 5	0.41	10 ± 3	10 ± 3	0.68
after	10 ± 6	12 ± 4	10 ± 2	11 ± 3		
change	1 ± 1	1 ± 2	<0.001	1 ± 2	2 ± 1	0.004
GEDVI, mL/m^2^						
baseline	1007 ± 306	870 ± 236	0.002	801 ± 186	834 ± 163	0.83
after	1102 ± 313	858 ± 208	872 ± 199	843 ± 167		
change	96 ± 59	−12 ± 54	<0.001	70 ± 85	8 ± 38	<0.001
PBVI, mL/m^2^						
baseline	215 ± 95	203 ± 64	0.25	212 ± 51	225 ± 50	0.86
after	250 ± 54	204 ± 52	224 ± 40	227 ± 50		
change	34 ± 63	1 ± 69	<0.001	11 ± 52	2 ± 53	<0.001
Fluid, mL	522 ± 120	467 ± 161	0.07	450 ± 176	467 ± 145	0.75
Gelatin/HES/	3/1/5	6/5/7	0.65	5/6/4	1/3/2	0.24
albumin						
**CI ≥15%**	R	NR	P-value	R	NR	P-value
	(n = 4 steps in 4 patients)	(n = 23 steps in 9 patients)		(n = 5 steps in 4 patients)	(n = 16 steps in 7 patients)	
CI, L/min/m^2^						
baseline	3.6 ± 1.0	3.6 ± 0.7	0.50	3.6 ± 1.3	4.9 ± 1.5	0.01
after	4.4 ± 1.1	3.7 ± 0	4.4 ± 1.5	5.0 ± 1.5		
change	0.9 ± 0.3	0.1 ± 0.3	na	0.8 ± 0.3	0 ± 0.2	na
CVP, mm Hg						
baseline	10 ± 4	11 ± 5	0.59	9 ± 3	10 ± 3	0.61
after	11 ± 4	12 ± 5	10 ± 3	11 ± 3		
change	1 ± 1	1 ± 1	0.76	1 ± 2	1 ± 1	0.05
GEDVI, mL/m^2^						
baseline	802 ± 214	935 ± 271	0.26	814 ± 205	829 ± 160	0.83
after	935 ± 254	940 ± 277	886 ± 219	841 ± 162		
change	133 ± 42	5 ± 63	<0.001	73 ± 96	12 ± 39	<0.001
PBVI, mL/m^2^						
baseline	159 ± 105	216 ± 67	0.17	213 ± 57	224 ± 48	0.68
after	218 ± 26	220 ± 60	215 ± 38	230 ± 50		
change	59 ± 87	4 ± 63	0.56	1 ± 51	6 ± 53	<0.001
Fluid, mL	600 ± 0	465 ± 153	<0.001	460 ± 195	462 ± 140	0.84
Gelatin/HES/	1/1/2	8/5/10	0.67	1/3/1	5/6/5	1.0
albumin						

Mean ± SD or number of patients where appropriate. Abbreviations: *R*, Responders, *N*R Non-responders, *CI* Cardiac index, *GEF* Global ejection fraction, *R* Responding fluid loading step, *NR* non-responding fluid loading step, *CVP* Central venous pressure, *GEDVI* Global end-diastolic volume index, *PBVI* Pulmonary blood volume index, *HES* Hydroxyethyl starch, n.a. not applicable.

### Correlations

**Figure 1:**
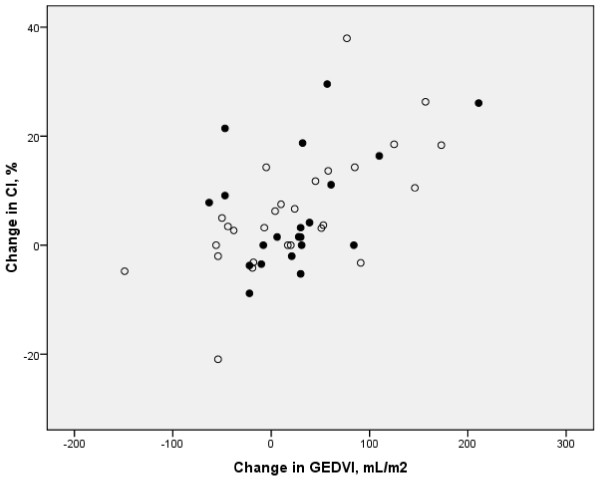
**Similar changes in cardiac index (CI,%) versus changes in global end-diastolic volume index (GEDVI, mL/m**^**2**^**) upon fluid loading steps in patients with low global ejection fraction (open circles, r = 0.65 P < 0.001) and those with near-normal global ejection fraction (closed circles, r = 0.42 P = 0.05) during severe sepsis or septic shock.**

Changes in PBVI did not correlate to changes in GEDVI and only the latter related to changes in CI, regardless of GEF (r = 0.56, P < 0.001; Figure 1). Changes between 0–90 min in SVI correlated to changes in GEDVI in the low GEF group only (r = 0.70, P = 0.03, n = 9).

## Discussion

Our study suggests that systolic cardiac dysfunction evidenced by a low GEF (29,30) is common in patients with severe sepsis or septic shock. This dysfunction occurring in 56% of our patients, independent of cardiac premorbidity, agrees with the literature [1-3,31]. Although this phenomenon might impair fluid responsiveness (9,18,24), our study suggests that fluid responsiveness can be maintained when the heart dilates, even during myocardial depression. In contrast, the optimum GEDVI in patients after cardiovascular surgery ranges from 680–800 mL/m^2^[34-36], and these values may thus not apply in sepsis. A maintained fluid responsiveness at higher GEDVI conforms to the idea that dilatation during sepsis-induced systolic dysfunction is as an adaptive response associated with survival by maintaining a relatively high CI [1-3,6,7,9,37]. Indeed, GEDVI was higher prior to responding than to non-responding steps according to CI ≥10% increases when GEF was low (7 of 9 [77%] responding steps had a baseline GEDVI >850 mL/m^2^). Also, it was not lower in responding than non-responding steps according to CI ≥15% increases, in contrast to the observations that a low baseline GEDVI, albeit dependent on GEF [25], is more often associated with fluid responsiveness than a relatively high GEDVI [21,26]. This confirms that the predictive value for fluid responsiveness of baseline GEDVI or end-diastolic dimensions, rather than changes, is imperfect by its dependency on systolic function, also in sepsis [21,26,27,38,39]. That the GEDVI prior to responding fluid loading steps was not lower compared to non-responding steps when GEF ≥20%, can be attributed to a difference in systolic function [26], since CI was lower in the latter. Finally, baseline GEDVI may depend on age and gender [40].

In contrast, we observed that patients with both systolic dysfunction and inability to dilate, were not fluid responsive. The inability to dilate upon systolic dysfunction could comply with the impaired relaxation and diastolic dysfunction found on echocardiography either as an isolated phenomenon or concomitant with systolic dysfunction in 20-60% of patients with severe sepsis or septic shock [10-16,25]. The phenomenon appeared associated with non-survival and was often transient and reversible in survivors. An additional hypothesis may be the presence of right ventricular dysfunction, in view of the increase in CVP. It cannot be excluded that the presence of predominant right ventricular dysfunction and dilatation limiting left ventricular filling though pericardial constraint may contribute to the lack of fluid responsiveness. Indeed, right ventricular dysfunction caused by moderate pulmonary hypertension (which was not monitored in this study) has been described to limit fluid responsiveness before [6,39]. Out data show that CVP increases upon fluid loading were slightly greater in non-responding than in responding steps which may also point to right ventricular dysfunction and dilatation in some of our patients with low GEF. However, in our study, the increase in CVP was also greater in non-responding than in responding fluid loading steps when GEF was near-normal, which may argue against predominant right ventricular dysfunction in non-responding fluid loading steps of low GEF patients. Since we did not perform operator-dependent, bedside echocardiography simultaneously, to differentiate between right or left ventricular dilatation, we cannot definitively decide on diastolic and/or right ventricular dysfunction in non-responding steps when GEF is low.

Patients with near-normal systolic function were also fluid-responsive by dilatation when operating in the steep part of the cardiac function curve. The dilatation associated with fluid responsiveness, as measured by an increase in GEDVI, is thus independent of systolic cardiac function. Our study partially agrees with data obtained by others suggesting that changes in filling pressures are less helpful in this respect than changes in GEDVI [21-23,26]. Apparently, the phenomenon that impaired systolic function renders filling pressures more important than volumes in the predictive and monitoring value of fluid responsiveness, while the opposite is true when systolic function is relatively normal, after cardiovascular surgery [33], may not apply to sepsis-induced cardiac dysfunction. Otherwise, a higher PEEP level applied in this series than in the previous one [27], may have contributed to the poor predictive value of CVP at low GEF.

Our study has some limitations. The number of patients is relatively small but the study was undertaken to improve interpretation of transpulmonary dilution data with fluid loading in severe sepsis and septic shock rather than to prove benefits thereof. The correlation between changes in GEDVI and CI, regardless of GEF, can be overestimated by mathematical coupling when both are derived from the same thermodilution curve, as argued before [41]. Since both PBVI and GEDVI are also derived, among others, from the same thermodilution curve, mathematical coupling with CI would affect both variables. That PBVI differed from GEDVI in responding to fluid loading and a rise in CI ≥15% and, in contrast to GEDVI, did not correlate to CI changes may, however, disfavor mathematical coupling. The CVP changes per step were relatively small in contrast to the overall changes over 90 min. Hence, the right ventricle may have been challenged enough to decide on cardiac responses to fluid loading. In our previous publications we also used hypotension/filling pressure criteria for defining clinical hypovolemia as a common trigger for fluid challenges [24,28,32]; we evaluated the predictive values for fluid responsiveness of other measurements later, to avoid confounding by giving fluids on the basis of allegedly superior predictors of fluid responsiveness than CVP. We used the delta CVP filling protocol as a safety measure rather than to predict fluid responsiveness. CVP starting values were adjusted for PEEP, in a relatively arbitrary manner, to roughly account for about 50% transmission of airway pressure, and CO responses were evaluated at two levels, even though the clinical relevance is unknown.

## Conclusions

In conclusion, our study suggests that in patients with sepsis-induced hypotension and systolic cardiac dysfunction, occurring in about half of patients, fluid responsiveness is maintained by global cardiac dilatation, as measured by transpulmonary dilution-derived GEDVI, rather than by an increase in CVP. Absence of fluid responsiveness in systolic cardiac dysfunction may be explained by diastolic dysfunction and/or concomitant right ventricular dysfunction. Transpulmonary (thermo)dilution-derived GEDVI is more helpful than CVP in monitoring fluid responsiveness and non-responsiveness and their mechanisms in sepsis-induced hypotension, but normal or targeted levels of preload (GEDVI 680–800 mL/m^2^) may not apply in this condition.

## Competing interests

The authors declare they have no competing interests.

## Authors’ contributions

RJT has made substantial contribution to the conception and design of the study including analysis and interpretation of the data as well as drafting the manuscript. ID has been involved in collecting and analysing the data. ABJG conceived of the study, participated in its design and coordination and helped to draft the manuscript and has given final approval of the version to be published. All authors read and approved the final manuscript.

## Pre-publication history

The pre-publication history for this paper can be accessed here:

http://www.biomedcentral.com/1471-2253/13/12/prepub

## References

[B1] Rabuel C Mebazaa A Septic shock: a heart story since the 1960s Intensive Care Med 2006 32 799 807 10.1007/s00134-006-0142-5 16570145

[B2] Hunter JD Doddi M Sepsis and the heart Br J Anaesthesia 2010 104 3 11 10.1093/bja/aep339 19939836

[B3] Jozwiak M Persichini R Monnet X Teboul JL Management of myocardial dysfunction in severe sepsis Semin Respir Crit Care Med 2011 32 206 214 10.1055/s-0031-1275533 21506057

[B4] Bouhemad B Nicolas-Robin A Arbelot C Arthaud M Féger F Rouby JJ Isolated and reversible impairment of ventricular relaxation in patients with septic shock Crit Care Med 2008 36 766 774 10.1097/CCM.0B013E31816596BC 18431265

[B5] Parker MM Shelhamer JH Bacharach SL Green MV Natanson C Frederick TM Damske BA Parrillo JE Profound but reversible myocardial depression in patients with septic shock Ann Intern Med 1984 100 483 490 10.7326/0003-4819-100-4-483 6703540

[B6] Schneider AJ Teule GJ Groeneveld AB Nauta J Heidendal GA Thijs LG Biventricular performance during volume loading in patients with early septic shock, with emphasis on the right ventricle: a combined haemodynamic and radionuclide study Am Heart J 1988 116 103 112 10.1016/0002-8703(88)90256-6 3394612

[B7] Parker MM McCarthy KE Ognibene FP Parrillo JE Right ventricular dysfunction and dilatation similar to left ventricular changes, characterize the cardiac depression of septic shock in humans Chest 1990 97 126 131 10.1378/chest.97.1.126 2295231

[B8] Landesberg G GIlon D Meroz Y Georgieva M Levin PD Goodman S Avidan A Beeri R Weisman C Jaffe AS Sprung CL Diastolic dysfunction and mortality in severe sepsis and septic shock Eur Heart J 2011 33 895 903 21911341 10.1093/eurheartj/ehr351 PMC3345552

[B9] Ognibene FP Parker MM Natanson C Shelhamer JH Parrillo JE Depressed left ventricular performance. Response to volume infusion in patients with sepsis and septic shock Chest 1988 93 903 910 10.1378/chest.93.5.903 3359845

[B10] Poelaert J Declerck C Vogelaers D Colardyn F Visser CA Left ventricular systolic and diastolic function in septic shock Intensive Care Med 1997 23 553 560 10.1007/s001340050372 9201528

[B11] Gödje O Peyerl M Seebauer T Dewald O Reichart B Reproducibility of double indicator dilution measurements of intrathoracic blood volume compartments, extravascular lung water, and liver function Chest 1998 113 1070 1077 10.1378/chest.113.4.1070 9554649

[B12] Jardin F Fourme T Page B Loubières Y Vieillard-Baron A Beauchet A Bourdarias JP Persistent preload defect in severe sepsis despite fluid loading: A longitudinal echocardiographic study in patients with septic shock Chest 1999 116 1354 1359 10.1378/chest.116.5.1354 10559099

[B13] Vieillard Baron A Schmitt JM Beauchet A Augarde R Prin S Page B Jardin F Early preload adaptation in septic shock? A transesophageal echocardiographic study Anesthesiology 2001 94 400 406 10.1097/00000542-200103000-00007 11374597

[B14] Etchecopar-Chevreuil C François B Clavel M Pichon N Gastinne H Vignon P Cardiac morphological and functional changes during early septic shock: a transesophageal echocardiographic study Intensive Care Med 2008 34 250 256 10.1007/s00134-007-0929-z 18004543

[B15] Bouhemad B Nicolas-Robin A Arbelot C Arthaud M Féger F Rouby J-J Acute left ventricular dilatation and shock-induced myocardial dysfunction Crit Care Med 2009 37 441 447 10.1097/CCM.0b013e318194ac44 19114917

[B16] Sturgess DJ Marwick TH Joyce C Jenkins C Jones M Masci P Stewart D Venkatesh B Prediction of hospital outcome in septic shock: a prospective comparison of tissue Doppler and cardiac biomarkers Crit Care 2010 14 R44 10.1186/cc8931 20331902 PMC2887156

[B17] Boyd JH Forbes J Nakada T Walley KR Russell JA Fluid resuscitation in septic shock: A positive fluid balance and elevated central venous pressure are associated with increased mortality Crit Care Med 2011 39 259 265 10.1097/CCM.0b013e3181feeb15 20975548

[B18] Weisel RD Vito L Dennis RC Valeri CR Hechtman HB Myocardial depression during sepsis Am J Surg 1977 133 512 521 10.1016/0002-9610(77)90141-6 848686

[B19] Packman MI Rackow E Optimum left heart filling pressure during fluid resuscitation of patients with hypovolemic and septic shock Crit Care Med 1983 11 165 169 10.1097/00003246-198303000-00003 6831886

[B20] Dellinger RP Levy MM Carlet JM Bion J Parker MM Jaeschke R Reinhart K Angus DC Brun-Buisson C Beale R Calandra T Dhainaut JF Gerlach H Harvey M Marini JJ Marshall J Ranieri M Ramsay G Sevransky J Thompson BT Townsend S Vender JS Zimmerman JL Vincent JL Surviving Sepsis Campaign: international guidelines for management of severe sepsis and septic shock: 2008 Crit Care Med 2008 36 296 327 10.1097/01.CCM.0000298158.12101.41 18158437

[B21] Marx G Cope T McCrossan L Swaraj S Cowan C Mostafa SM Wenstone R Leuwer M Assessing fluid responsiveness by stroke volume variation in mechanically ventilated patients with severe sepsis Eur J Anaesthesiol 2004 21 132 138 14977345 10.1017/s0265021504002091

[B22] Marik PE Baram M Vahid B Does central venous pressure predict fluid responsiveness? A systematic review of the literature and the tale of seven mares Chest 2008 134 172 178 10.1378/chest.07-2331 18628220

[B23] Scheuren K Wente MN Hainer C Scheffler M Lichtenstern C Martin E Schmidt J Bopp C Weigand MA Left ventricular end-diastolic area is a measure of cardiac preload in patients with early septic shock Eur J Anaesthesiol 2009 26 759 765 10.1097/EJA.0b013e32832a3a9c 19390446

[B24] Trof RJ Sukul SP Twisk JW Girbes AR Groeneveld AB Greater cardiac response of colloid than saline fluid loading in septic and non-septic critically ill patients with clinical hypovolaemia Intensive Care Med 2010 36 697 701 10.1007/s00134-010-1776-x 20165941 PMC2837190

[B25] Hofer CK Furrer L Matter-Ensner S Malpogne M Klaghofer R Genoni M Zollinger A Volumetric preload measurement by thermodilution: a comparison with transoesophageal echocardiography Br J Anaesthesia 2005 94 748 755 10.1093/bja/aei123 15790674

[B26] Michard F Alaya S Zarka V Bahloul M Richard C Teboul JL Global end-diastolic volume as an indicator of cardiac preload in patients with septic shock Chest 2003 124 1900 1908 10.1378/chest.124.5.1900 14605066

[B27] Malbrain ML De Potter TJ Dits H Reuter DA Global and right ventricular end-diastolic volumes correlate better with preload after correction for ejection fraction Acta Anaesthesiol Scand 2010 54 622 631 10.1111/j.1399-6576.2009.02202.x 20085545

[B28] Van der Heijden M Verheij J Van Nieuw Amerongen GP Groeneveld ABJ Crystalloid or colloid fluid loading and pulmonary permeability, edema and injury in septic and non-septic critically ill patients with hypovolemia Crit Care Med 2009 37 1275 1281 10.1097/CCM.0b013e31819cedfd 19242338

[B29] Combes A Berneau J-B Luyt C-E Trouillet J-L Estimation of left ventricular systolic function by single transpulmonary thermodilution Intensive Care Med 2004 30 1377 1383 15105983 10.1007/s00134-004-2289-2

[B30] Jabot J Monnet X Bouchra L Chemla D Richard C Teboul J-L Cardiac function index provided by transpulmonary thermodilution behaves as an indicator of left ventricular systolic function Crit Care Med 2009 37 2913 2918 10.1097/CCM.0b013e3181b01fd9 19866507

[B31] Ritter S Rudiger A Maggiorini M Transpulmonary thermodilution-derived cardiac function index identifies cardiac dysfunction in acute heart failure and septic patients: an observational study Crit Care 2009 13 R133 10.1186/cc7994 19671146 PMC2750191

[B32] Trof RJ Danad I Reilingh MW Breukers RM Groeneveld AJ Cardiac filling volumes versus pressures for predicting fluid responsiveness after cardiovascular surgery: the role of systolic cardiac function Crit Care 2011 15 R73 10.1186/cc10062 21352541 PMC3222006

[B33] Levy MM Fink MP Marshall JC Abraham E Angus D Cook D Cohen J Opal SM Vincent JL Ramsay G SCCM/ESICM/ACCP/ATS/SIS 2001: **SCCM/ESICM/ACCP/ATS/SIS International Sepsis Definitions Conference** Crit Care Med 2003 31 1250 1256 10.1097/01.CCM.0000050454.01978.3B 12682500

[B34] Goepfert MS Reuter DA Akyol D Lamm P Kilger E Goetz AE Goal-directed fluid management reduces vasopressor and catecholamine use in cardiac surgery patients Intensive Care Med 2007 33 96 103 10.1007/s00134-006-0404-2 17119923

[B35] Carl M Alms A Braun J Dongas A Erb J Goetz A Goepfert M Gogarten W Grosse J Heller AR Heringlake M Kastrup M Kroener A Loer SA Marggraf G Markewitz A Reuter D Schmitt DV Schirmer U Wiesenack C Zwissler B Spies C S3 guidelines for intensive care in cardiac surgery patients: hemodynamic monitoring and cardiocirculatory system Ger Med Sci 2010 8 1 25 10.3205/000101 PMC2890209 20577643

[B36] Kirov MY Kuzkov VV Molnar Z Perioperative haemodynamic therapy Curr Opin Crit Care 2010 16 384 392 10.1097/MCC.0b013e32833ab81e 20508520

[B37] Eichhorn V Goepfert MS Eulenburg C Malbrain ML Reuter DA Comparison of values in critically ill patients for global end-diastolic volume and extravascular lung water measured by transcardiopulmonary thermodilution: A metaanalysis of the literature Med Intensiva 2012 36 467 474 10.1016/j.medin.2011.11.014 22285070

[B38] Feissel M Michard F Mangin I Ruyer O Faller JP Teboul JL Respiratory changes in aortic blood velocity as an indicator of fluid responsiveness in ventilated patients with septic shock Chest 2001 119 867 877 10.1378/chest.119.3.867 11243970

[B39] Wyler von Ballmoos M Takala J Roeck M Porta F Tueller D Ganter CC Schröder R Bracht H Baenziger B Jakob SM Pulse-pressure variation and hemodynamic response in patients with elevated pulmonary artery pressure: a clinical study Crit Care 2010 14 R111 10.1186/cc9060 20540730 PMC2911757

[B40] Wolf S Riess A Landscheidt JF Lumenta CB Friederich P Schürer L Global end-diastolic volume acquired by transpulmonary thermodilution depends on age and gender in awake and spontaneously breathing patients Crit Care 2009 13 R202 10.1186/cc8209 20003415 PMC2811898

[B41] Breukers RM de Wilde RB van den Berg PC Jansen JR Faes TJ Twisk JW Groeneveld AB Assessing fluid responses after coronary surgery: role of mathematical coupling of global end-diastolic volume to cardiac output measured by transpulmonary thermodilution Eur J Anaesthesiol 2009 26 954 960 10.1097/EJA.0b013e32833098c6 19652601

